# Impact of HR-HPV infection on oncological outcomes in early cervical cancer

**DOI:** 10.3389/fonc.2023.1264114

**Published:** 2023-08-28

**Authors:** Xiaoqiang Su, Pan Liu, Hongwei Zhao, Lixin Sun, Wuliang Wang, Shuanglin Jin, Hui Wang, Ping Liu, Chunlin Chen, Min Hao

**Affiliations:** ^1^ Department of Obstetrics and Gynecology, The Second Hospital of Shanxi Medical University, Taiyuan, China; ^2^ Department of Obstetrics and Gynecology, Nanfang Hospital, Southern Medical University, Guangzhou, China; ^3^ Department of Gynecologic Oncology, Shanxi Tumor Hospital, Taiyuan, China; ^4^ Department of Obstetrics and Gynecology, The Second Affiliated Hospital of He’ nan Medical University, Zhengzhou, China; ^5^ Department of Obstetrics and Gynecology, Peace Hospital Affiliated to Changzhi Medical College, Changzhi, China

**Keywords:** cervical neoplasms, HR-HPV negative group, HR-HPV positive group, real-world study, oncological outcomes

## Abstract

**Background:**

This study aimed to investigate the differences in long-term oncological outcomes between high-risk human papillomavirus (HR-HPV) negative and HR-HPV positive early-stage cervical cancers.

**Methods:**

We retrospectively analysed 2061 cases of early-stage cervical cancer from the Chinese cervical cancer clinical diagnosis and treatment database. Kaplan-Meier curves were used to describe the survival outcomes of different HR-HPV infections. Cox proportional hazard regression model was used to analyze and determine independent risk factors.

**Results:**

K-M analysis revealed no significant difference in 5-year OS between HR-HPV negative and HR-HPV positive groups (OS: 95.0% vs.95.6%, P=0.900). A significant difference was observed in 5-year DFS between the HR-HPV negative and HR-HPV positive groups (DFS: 87.2% vs.91.9%, P=0.025). Cox proportional hazard regression model indicated that HR-HPV infection (negative vs. positive) was an independent factor influencing 5-year DFS after early cervical cancer surgery (DFS: hazard ratio [HR]=1.862, P=0.022). HR-HPV infection (negative vs positive) was not an independent factor influencing 5-year OS after early cervical cancer surgery (OS: P=0.813). After 1:1 PSM pairing, there was no significant difference in 5-year OS and DFS between HR-HPV negative group and HR-HPV positive group (OS: 91.6% vs.95.0%, P=0.297; DFS: 87.2% vs.85.1%, P=0.758). Cox multivariate analysis indicated that HR-HPV infection was not an independent factor influencing 5-year OS and DFS after early cervical cancer surgery (OS: P=0.806, DFS: P=0.251).

**Conclusions:**

The tumour results of HR-HPV negative group and HR-HPV positive group were similar, after eliminating the differences in known variables that affect the oncological outcomes of cervical cancer. The treatment plan of HR-HPV positive cervical cancer is suitable for HR-HPV negative cervical cancer.

## Introduction

Cervical cancer is the fourth most common malignant tumor that threatens women’s health worldwide. According to data from the International Agency for Research on Cancer, it is estimated that there will be approximately 604,000 new cases and 342,000 deaths due to cervical cancer globally in 2020. In low-income developing countries and regions, the number of new cases and deaths due to cervical cancer ranks second among female malignant tumor (1). Notably, etiological research on cervical cancer has seen a series of breakthroughs. In the 1980s, German virologist Harald Zurhausen proposed that high-risk human papillomavirus (HR-HPV) infection is closely associated with cervical cancer (2). Epidemiological investigations have confirmed that HR-HPV is detectable in 95–99% of cervical cancer tissues (3). With the further research on cervical cancer pathogenesis, the long-term persistent infection of HR-HPV is the decisive factor leading to the occurrence and development of cervical cancer. However, the recent study of 209 cases of cervical cancer in Sweden shows that 7% of tumor patients are still HPV negative using three different methods of genotyping and the reassessment of tumor materials by pathologists (4). In 2019, Malin et al. showed that the use of alternative methods and viral targets for extended analysis of HPV negative cervical cancer patients can reduce the HPV negative proportion from 14% to 7% (5). In clinical practice, with no matter what detection method, some patients with cervical cancer are still not found to have HR-HPV infection. However, the etiology and pathogenesis of these patients are not very clear, and the tumor outcome is rarely reported after clinical treatment. To address these gaps in the field, we compared and analyzed oncologic outcomes of open surgery in HR-HPV-negative and HR-HPV positive cases of stage IA1–IIA2 cervical cancer in real-world settings. To this end, we harnessed data on 63926 cases from databases of 37 hospitals in mainland China in order to elucidate the prognosis of patients with stage I A1–II A2 cervical cancer undergoing laparotomy.

## Methods

### Data sources

This study was a multicentre, retrospective, observational study, a cervical cancer specialized disease database (n=63926) that covers consecutive patients with cervical cancer in 37 hospitals in mainland China treated since January 2004. The Southern Hospital Ethics Committee of Southern Medical University reviewed the establishment of the cervical cancer database (Ethics No. NFEC-2017-135). The identifier of the clinical trial is CHiCTR180017778 (International Clinical Trials Registry Platform Search Port, http://apps.who.int/trialsearch/).

Clinical data were collected from patient files and the medical record management system in the hospitals by trained gynaecological oncology staff using standardized data collection and quality control procedures. The details of the data sources and methods were the same as those previously reported (6–8). For patients underwent surgical treatment, the collected data contained almost all the information during the treatment of cervical cancer, including demographic details, preoperative examination results, surgical information, pathological results, preoperative and postoperative adjuvant treatment details, complications, hospitalization time and expenses, and follow-up. To ensure the accuracy of the collected data, two uniformly trained staff used EpiData software (EpiData Association, Odense M, Denmark) to input and proofread the same data from each hospital.

All follow-up procedures were carried out by trained gynaecological oncology staff at each centre to keep the patients’ personal data confidential and to simultaneously provide disease management guidance. Follow-up information, including the survival status, time of death, recurrence time, recurrence site, and treatment after recurrence, was gathered through the return visit system or through a telephone follow-up. Vaginal stump recurrence was usually confirmed by pathological biopsy, abdominal and pelvic recurrence is detected by computer tomography (CT) or magnetic resonance imaging (MRI), and a few patients are detected by positron emission tomography-CT. The oncological outcomes were estimated according to the recorded information, and the last day of the return visit or telephone follow-up was defined as the last follow-up. In this database, the final International Federation of Gynecology and Obstetrics (FIGO) stage was corrected by tumor size according to the FIGO 2018 staging system. Tumor size was determined by final pathological records.

### Inclusion and exclusion criteria

Entry conditions and grouping were as follows (1): Chinese female, age ≥ 18 years; (2) FIGO stage included IA1 (lymphatic vascular space infiltration (LVSI)-positive) - IIA2 stage (including unknown sub-stages of IA (LVSI-positive), IB, IIA); (3) histological type was squamous cell carcinoma, adenocarcinoma, and adeno-squamous cell carcinoma; (4) no preoperative adjuvant therapy was administered; (5) surgical approach was laparotomy; (6) operation method: IA1 (LVSI-positive), IA (LVSI-positive), and IA2 patients underwent QM-B type surgery, while the remaining patients underwent QM-C type surgery; (7) survival outcomes were available; (8) Availability of HR-HPV status. The exclusion criteria were as follows: (1) accidental discovery of cervical cancer, pregnancy complicated by cervical cancer, stump cancer, and other types of malignant tumors concurrently; (2) patients who did not meet the inclusion criteria.

### Definition

The staging rules for cervical cancer in FIGO 2018 are based on the combination of clinical imaging and pathological diagnosis results. The following four points should be noted for staging: 1. Two or more senior physicians should conduct a joint physical examination to clarify the clinical staging. When conditions permit, it is best to perform pelvic examination under anesthesia. 2. When there are differences in stages, the earlier stage shall prevail. 3. Allow imaging and pathological examination results to be used for staging. 4. The diagnosis of minimally invasive carcinoma must be made by an experienced pathologist based on cervical conization specimens.

In this study, all patients were tested for HPV by in-house polymerase chain reaction (PCR). For cervical cancer patients with negative HR-HPV in the first screening, the second sampling and testing were conducted by the same method. Patients who tested negative twice were classified as HR-HPV negative patients.

The 5-year DFS was defined as the date from the operation to the date of death due to cervical cancer or recurrence of cervical cancer. OS was defined as the date from the operation to the date of death from any cause. Patients with no evidence of recurrence or death were defined by the date of the last follow-up date or the last outpatient visit.

### Statistical analysis

Continuous variables are summarized by means ± standard deviation, while count variables are summarized by frequency and percentage. The comparison between the mean values of continuous variables is conducted using independent sample t-tests, and the comparison between the rates of counting data groups adopts χ 2 Test, rank variable adopts nonparametric rank sum test. The t-test and the χ 2 Test were used to analyze the clinical pathological characteristics and differences between the HR-HPV negative group and the HR-HPV positive group in early cervical cancer populations. The statistical software used was Statistical Product and Service Solutions 23.0 (SPSS, Inc., Chicago, IL, USA). The P-value <0.05 was considered statistically significant.

Kaplan-Meier curves were used to describe the survival outcomes of different HR-HPV infections. Cox proportional hazard regression model was used to analyze and determine independent risk factors, and estimate the hazard ratio (HR) and 95% confidence interval (CI) of the impact of HR-HPV infection on the 5-year OS and DFS rates. In the Cox proportional risk regression models, we included clinical variables regarded as known factors affecting the oncological outcomes of cervical cancer (age, histological type, FIGO stage, tumor diameter, depth of cervical invasion, LVSI, Parametrial invasion, vaginal margin, and postoperative adjuvant therapy).

In the propensity score matching (PSM) analysis, patients in the HR-HPV negative group were matched to patients in the HR-HPV positive group based on propensity score to reduce bias. Then, a new group of patients was constructed with different HR- HR-HPV infection but similar other clinicopathological features. The propensity score of each patient to receive HR-HPV negative patients was calculated by logistic regression model, which included clinical variables of known factors affecting the oncological outcomes of cervical cancer (age, histological type, FIGO stage, tumor diameter, depth of cervical invasion, LVSI, parametrial invasion, vaginal margin, and postoperative adjuvant therapy). This propensity score was used for one-to-one matching cases with the nearest neighbor matching with variance of 0.02.

## Results

**Figure 1:**
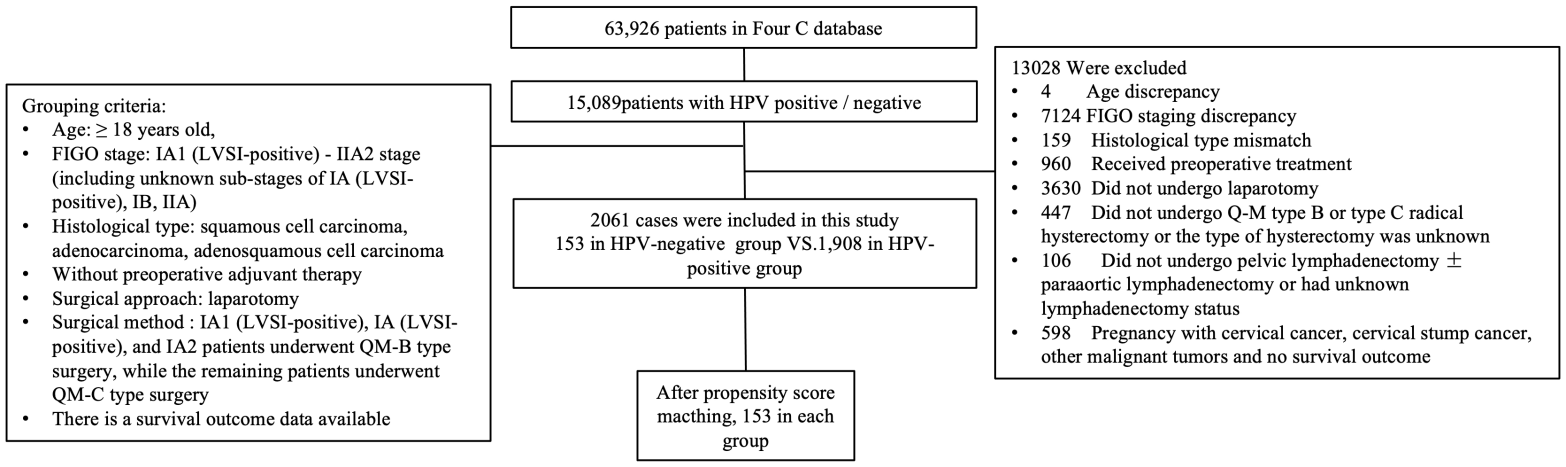
Flow diagram of recruitment and exclusions. HPV, human papillomavirus; FIGO, Federation International of Gynecology and Obstetrics; QM, Querleu-Morrow.

A total of 2,061 cases met the enrolment criteria. The detailed data-filtering process is presented in **Figure 1**.

Comparison of oncological outcomes between HR-HPV negative and HR-HPV positive surgical cases of early cervical cancer 2061 cases of cervical cancer in IA1~IIA2 stage met the initial inclusion criteria, including 153 cases in HR-HPV negative group and 1908 cases in HR-HPV negative group (**Table 1**).

**Table 1:** The clinicopathologic characteristics of patients in HPV-positive group and HPV-negative group before matching.

Characteristics	HPV- positive (n=1908)		HPV-negative (n=153)		P value
Age	47.88 ± 9.839		47.58 ± 9.397		0.716
Histological type					<0.001
Squamous cell carcinoma	1711	89.70%	121	79.10%	
Adenocarcinoma	157	8.20%	30	19.60%	
Adenosquamous carcinoma	40	2.10%	2	1.30%	
FIGO stage					0.357
IA1	39	2.00%	1	0.70%	
IA2	51	2.70%	8	5.20%	
IB1	479	25.10%	36	23.50%	
IB2	726	38.10%	49	32.00%	
IIA1	408	21.40%	37	24.20%	
IIA2	89	4.70%	11	7.20%	
IA	55	2.90%	3	2.00%	
IB	33	1.70%	4	2.60%	
IIA	20	1.00%	3	2.00%	
I	7	0.40%	1	0.70%	
II	1	0.10%	0	0.00%	
Tumor diameter					0.276
≤4cm	1731	90.70%	133	86.90%	
>4cm	89	4.70%	11	7.20%	
Unreported	88	4.60%	9	5.90%	
Depth of cervical invasion					0.869
≤1/2	894	46.90%	69	45.10%	
>1/2	835	43.80%	68	44.40%	
Unreported	179	9.40%	16	10.50%	
LVSI					0.812
Negative	1620	84.90%	131	85.60%	
Positive	288	15.10%	22	14.40%	
Parauterine infiltration					0.306
Negative	1895	99.30%	153	100.00%	
Positive	13	0.70%	0	0.00%	
Vaginal margin					0.910
Negative	1868	97.90%	150	98.04%	
Positive	40	2.10%	3	1.96%	
Postoperative adjuvant therapy					0.079
None	888	46.50%	71	46.40%	
Chemotherapy	268	14.00%	12	7.80%	
Radiotherapy	286	15.00%	22	14.40%	
Radiotherapy/radiochemotherapy	466	24.40%	48	31.40%	

Values are presented as mean ± standard deviation or number (%). Bold indicates significant p-value.

LVSI, lymphovascular space invasion.

**Figure 2:**
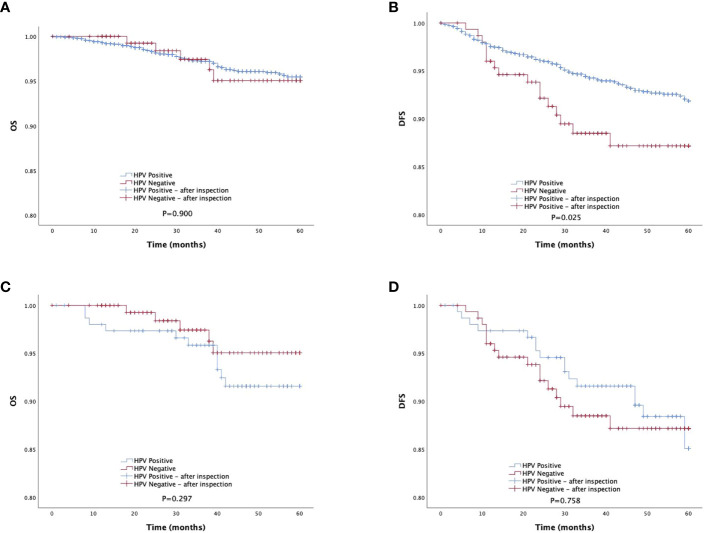
Survival outcomes between HPV-negative group and HPV-positive group in study population. DFS, disease-free survival; PSMpropensity score matching. **(A, B)** The 5-year DFS and OS of total study population. **(C, D)** The 5-year DFS and OS of total study population after PSM matching.

The survival analysis revealed no significant difference in 5-year OS (OS: 96.7% vs.96.9%, P=0.900) between the HR-HPV-negative and HR-HPV positive groups, but there was a significant difference between the HR-HPV negative group and the HR-HPV positive group in the 5-year DFS (DFS: 89.5% vs.94.0%, P=0.025) (**Figures 2A, B**).

Cox multivariate analysis indicated that HR-HPV infection (negative vs. positive) was not an independent factor influencing 5-year postoperative death due to early cervical cancer (OS: P = 0.813) (**Table 2**). HR-HPV infection (negative vs. positive) is an independent influencing factor for recurrence/death of early cervical cancer 5 years after surgery (DFS: P=0.022) (**Table 2**). HR-HPV positive is a risk factor for DFS 5 years after surgery. The risk of recurrence/death in HR-HPV positive group is 1.862 times that in negative group.

**Table 2:** Association of HPV infection and survival in cervical cancer by multivariable analysis.

Characteristics	OS				DFS			
	P	HR	95.0% CI		P	HR	95.0% CI	
Age	0.341	1.012	0.987	1.038	0.873	1.001	0.984	1.020
HPV	0.813	1.117	0.444	2.810	0.022	1.862	1.095	3.166
FIGO stage								
IA1	0.933				0.821			
IB1	0.895	2802.410	0.000	8.476E+54	0.868	5819.890	0.000	1.119E+48
IB2	0.889	4529.725	0.000	1.377E+55	0.862	8530.113	0.000	1.640E+48
IIA1	0.884	6278.068	0.000	2.009E+55	0.858	10687.868	0.000	2.055E+48
IIA2	0.893	3168.755	0.000	1.011E+55	0.867	6201.489	0.000	1.196E+48
IA2	1.000	1.037	0.000	9.009E+83	0.999	1.141	0.000	7.298E+70
IA	0.998	0.779	0.000	8.817E+90	0.869	5490.128	0.000	1.075E+48
IB	0.944	7435.606	0.000	1.244E+112	0.826	35659413.996	0.000	8.880E+74
IIA	0.997	0.527	0.000	1.128E+132	0.821	58772346.686	0.000	1.465E+75
I	0.999	0.866	0.000	4.375E+164	0.991	4.502	0.000	7.015E+110
II	1.000	1.073	0.000	.	0.999	0.785	0.000	.
Histological type								
Squamous cell carcinoma	0.617				0.885			
Adenocarcinoma	0.654	0.782	0.266	2.300	0.715	1.123	0.603	2.092
Adenosquamous carcinoma	0.400	1.855	0.440	7.814	0.721	1.235	0.387	3.938
Tumor diameter								
≤4cm	0.989				0.991			
>4cm	0.947	0.961	0.301	3.069	0.914	0.956	0.421	2.171
Unreported	0.900	1.067	0.387	2.944	0.929	0.966	0.450	2.073
Depth of cervical invasion								
≤1/2	0.000				0.000			
>1/2	0.000	3.700	1.923	7.116	0.000	2.550	1.667	3.901
Unreported	0.361	0.387	0.051	2.961	0.127	0.399	0.123	1.297
LVSI	0.102	1.661	0.904	3.050	0.043	1.582	1.014	2.469
Parauterine infiltration	0.266	2.387	0.515	11.055	0.132	2.534	0.756	8.491
Vaginal margin	0.560	0.553	0.075	4.059	0.586	1.326	0.481	3.656
Postoperative adjuvant therapy								
None	0.075				0.009			
Chemotherapy	0.081	0.513	0.242	1.087	0.042	0.569	0.330	0.981
Radiotherapy	0.350	0.691	0.318	1.501	0.225	0.721	0.424	1.224
Radiotherapy/radiochemotherapy	0.014	0.442	0.231	0.847	0.001	0.450	0.280	0.724

Multicollinearity test and cox proportional hazard regression models were used for analysis. Bold indicates significant p-value.

CI, confidence interval; DFS, disease-free survival; HR, hazard ratio; LVSI, lymphovascular space invasion; OS, overall survival.

Comparison of oncological outcomes between HR-HPV-negative and HR-HPV positive surgical cases of early cervical cancer after further enrolment and matching.

Meet the initial inclusion criteria and strictly follow the histological type, LVSI, postoperative adjuvant therapy 1:1 matching. The matching tolerance is 0, including 153 cases each in the HR-HPV positive and HR-HPV negative group (**Table 3**).

**Table 3:** The clinicopathologic characteristics of patients in HPV-positive group and HPV-negative group after matching.

Characteristics	HPV-positive (n=153)		HPV-negative (n=153)		P value
Age	46.48 ± 9.210		47.58 ± 9.397		0.303
Histological type					1.000
Squamous cell carcinoma	121	79.10%	121	79.10%	
Adenocarcinoma	30	19.60%	30	19.60%	
Adenosquamous carcinoma	2	1.30%	2	1.30%	
FIGO stage					0.421
IA1	9	5.90%	8	5.20%	
IA2	1	0.70%	1	0.70%	
IB1	38	24.80%	36	23.50%	
IB2	65	42.50%	49	32.00%	
IIA1	30	19.60%	37	24.20%	
IIA2	4	2.60%	11	7.20%	
IA	1	0.70%	3	2.00%	
IB	2	1.30%	4	2.60%	
IIA	2	1.30%	3	2.00%	
I	0	0.00%	1	0.70%	
II	1	0.70%	0	0.00%	
Tumor diameter					0.089
≤4cm	144	94.10%	133	86.90%	
>4cm	4	2.60%	11	7.20%	
Unreported	5	3.30%	9	5.90%	
Depth of cervical invasion					0.592
≤1/2	70	45.80%	69	45.10%	
>1/2	72	47.10%	68	44.40%	
Unreported	11	7.20%	16	10.50%	
LVSI					1.000
Negative	131	85.60%	131	85.60%	
Positive	22	14.40%	22	14.40%	
Parauterine infiltration					0.082
Negative	150	98.00%	153	100.00%	
Positive	3	2.00%	0	0.00%	
Vaginal margin					0.474
Negative	148	96.70%	150	98.00%	
Positive	5	3.30%	3	2.00%	
Postoperative adjuvant therapy					1.000
None	71	46.40%	71	46.40%	
Chemotherapy	12	7.80%	12	7.80%	
Radiotherapy	22	14.40%	22	14.40%	
Radiotherapy/radiochemotherapy	48	31.40%	48	31.40%	

Values are presented as mean± standard deviation or number (%). Bold indicates significant p-value.

LVSI, lymphovascular space invasion.

The survival analysis showed that there was no statistically significant difference between the HR-HPV negative and the HR-HPV positive group in the 5-year OS (OS: 96.7% vs.92.8%, P=0.297), and there was no statistically significant difference between the HR-HPV negative and the HR-HPV positive group in the 5-year DFS (DFS: 89.5% vs.88.9%, P=0.758) (**Figures 2C, D**). Cox multifactor analysis showed that HR-HPV infection (negative vs positive) was not an independent factor (OS: P=0.806) influencing 5-year mortality after surgery for early cervical cancer (**Table 4**), and influencing factor for recurrence/death of early cervical cancer 5 years after surgery (DFS: P=0.251) (**Table 4**).

**Table 4:** Association of HPV infection and survival in cervical cancer by multivariable analysis after PSM matching.

Characteristics	OS				DFS			
	P	HR	95.0% CI		P	HR	95.0% CI	
Age	0.114	1.046	0.989	1.107	0.294	1.021	0.982	1.061
HPV	0.806	0.869	0.282	2.672	0.251	1.529	0.741	3.156
FIGO stage								
IA1	0.997				0.987			
IB1	0.917	198.284	0.000	2.217E+45	0.858	967.793	0.000	4.408E+35
IB2	0.889	1184.989	0.000	1.296E+46	0.847	1651.144	0.000	7.505E+35
IIA1	0.887	1284.564	0.000	1.406E+46	0.849	1480.011	0.000	6.735E+35
IIA2	0.991	0.416	0.000	3.813E+63	0.987	0.400	0.000	8.542E+47
IA2	0.997	0.634	0.000	1.346E+113	0.997	0.695	0.000	9.624E+79
IA	0.985	20.476	0.000	1.384E+137	0.986	7.253	0.000	9.531E+95
IB	0.962	67647.126	0.000	8.265E+204	0.954	16614.497	0.000	9.451E+146
IIA	0.991	18.993	0.000	1.643E+215	0.946	90243.195	0.000	5.119E+147
I	0.973	33925.276	0.000	1.156E+269	0.965	18234.065	0.000	2.416E+192
II	0.996	0.393	0.000	1.725E+175	0.997	0.610	0.000	2.369E+124
Histological type								
Squamous cell carcinoma	0.563				0.389			
Adenocarcinoma	0.284	2.063	0.548	7.760	0.259	1.666	0.686	4.048
Adenosquamous carcinoma	0.954	0.002	0.000	1.619E+92	0.340	2.959	0.319	27.416
Tumor diameter								
≤4cm	0.992				0.660			
>4cm	0.980	0.000	0.000	.	0.971	0.000	0.000	3.919E+272
Unreported	0.902	1.135	0.150	8.595	0.362	1.738	0.530	5.704
Depth of cervical invasion								
≤1/2	0.294				0.241			
>1/2	0.121	3.226	0.735	14.156	0.096	2.074	0.879	4.895
Unreported	0.857	0.002	0.000	2.424E+27	0.790	0.001	0.000	1.967E+20
LVSI	0.715	0.766	0.184	3.200	0.012	9.984	1.648	60.493
Parauterine infiltration	0.009	13.453	1.891	95.707	0.015	9.239	1.550	55.079
Vaginal margin	0.580	1.964	0.180	21.406	0.782	0.722	0.071	7.294
Postoperative adjuvant therapy								
None	0.470				0.500			
Chemotherapy	0.743	1.293	0.278	6.018	0.694	1.286	0.368	4.493
Radiotherapy	0.543	0.554	0.083	3.707	0.515	1.425	0.491	4.134
Radiotherapy/radiochemotherapy	0.211	0.409	0.101	1.661	0.348	0.624	0.233	1.672

Multicollinearity test and cox proportional hazard regression models were used for analysis. Bold indicates significant p-value.

CI, confidence interval; DFS, disease-free survival; HR, hazard ratio; LVSI, lymphovascular space invasion; OS, overall survival; PSM, propensity score matching.

## Discussion

In this study, our previous study showed that HR-HPV infection (negative vs. positive) is an independent influencing factor for recurrence/death of early cervical cancer 5 years after surgery. However, after PSM matching to eliminate relevant confounders, we found that HPV infection was not an independent influencer of recurrence/death after early cancer surgery.

This study was based on the real conditions in some parts of Chinese Mainland. in order to explore the impact of HR-HPV infection on the oncological outcome of early cervical cancer after laparotomy. The subjects were patients with stage IA1~IIA1 cervical cancer treated by laparotomy. This study was a multicenter study based on the real-world study, covering a large database of 63926 cases in 37 hospitals of different regions, levels and categories in China. It can reflect the real research situation of oncological outcomes of IA1~IIA1 cervical cancer patients with different HR-HPV infection in China after laparotomy.

At present, studies have confirmed that cervical cancer is caused by HR-HPV infection. persistent infection with HR-HPV (especially type 16) can cause cancer of the cervix (9). HPV plays an important role in the pathogenesis of cervical cancer. It affects host cell apoptosis, cell cycle, cell adhesion and DNA repair mechanisms, and can also activate immune response (10, 11). In addition, the integration of HR-HPV virus is closely related to the development of cervical cancer (12). HR-HPV also affects the prognosis of cervical cancer.

However, several recent studies have shown that HR-HPV infection has a paradoxical impact on the prognosis of cervical cancer. Liana et al. believe that HPV-negative cervical cancer patients were significantly more likely to have adverse outcomes than HPV 16/18-positive patients (P=0.018; OR=3.31) (13). Ping Li et al. believed that HPV-DNA positive status was associated with good prognosis in patients with cervical cancer (OS: HR=0.610, 95% CI=0.457-0.814, P=0.001; DFS: HR=0.362, 95% CI=0.252-0.519, P < 0.001) (14). Go et al. suggested that DFS of HPV-negative cervical cancer patients was worse than that of HPV positive ones (HR=3.97; 95% CI=1.84-8.58; P=0.0005) (15). Many other publications have reported that the DFS of HPV-negative cervical cancer patients after radiotherapy or chemotherapy is low regardless of other prognostic factors (age, stage, lymph node metastasis) (16–18). In other HPV related tumor studies, Anthony et al. believed that OS and DFS of HPV positive tumor patients were improved in 3 years compared with HPV negative tumor patients in oropharyngeal squamous cell carcinoma (90% vs 65%, respectively, P=0.001; 85% vs 49%, P=0.005) (19).

There are still some reports suggesting that there is no significant correlation between HPV infection and tumor prognosis. A recent systematic study found that there was no statistically significant association between HPV16 and/or HPV18 positive and overall survival or disease-free survival of cervical cancer (20). In a study of adeno-squamous carcinoma of the head and neck, Giacomo et al. suggested that HPV positive and HPV-negative tumors had similar OS and DFS (21). These findings support the present study.

With the further study of cervical cancer, the relationship between HPV infection and prognosis has been changing. HPV infection is a decisive factor in the occurrence of cervical cancer, but in actual clinical work, a small number of cervical cancer patients have negative HPV detection. HPV negative squamous cervical carcinoma is very rare. HPV positivity of among adenosquamous cancers (ADS) may be up to 86%, the prevalence of HPV among adenocarcinoma (ADC) varies between the subtypes (Usual type 80-100%; Mucinous, Intestinal type 83-100%; Villoglandular 100%; Mucinous, signet ring cell type 100%; Endometrioid 0; Gastric Type 0; Masonephric 0; Clear cell 28%; Serous 30%) (22, 23). The pathogenesis of non HPV-associated adenocarcinoma(NHPVA) is considered irrelevant or independent of HPV (24). In fact, NHPVA is related to mutation. As for tumor inhibitor p53, the loss of its function due to the change of TP53 gene is a common event of cancer in different anatomical regions. Barreto et al. showed that there was a relationship between p53 mutation and poor prognosis (24). In Nicolás et al.’s study, 71% (15/21) HPV negative patients had p53abn (25). This mutation phenotype of NHPVA can explain that the tumor has higher relaxation and regulation ability, increased growth potential and metastasis, and worse prognosis. Other scholars’ studies suggest that HR-HPV negative tumors may have become permanent and lost internal mutation control, so that somatic host mutations related to malignant growth and diffusion potential are obtained, while HR-HPV positive tumors may be better controlled by the immune system due to the expression of viral proteins, so the prognosis is relatively more positive (26).

In order to avoid the influence of different pathological tissue types, LVSI, and postoperative adjuvant therapy on the tumor outcome of cervical cancer patients as much as possible, this study strictly controls them to eliminate the influence of the above differences on the tumor outcome of cervical cancer patients. However, there are still limitations of HPV detection in clinical practice. Sampling errors may be the primary cause of false negative HPV testing. For example, low cellularity (due to cancer necrosis and/or inflammation), influence of blood or lubricants, cell fixation or cell lysis may lead to classification errors. It is reported that the use of formalin fixed and paraffin embedded samples had an impact on DNA preservation and subsequent HPV-DNA test results, leading to the high prevalence of HPV negative tumors (24). The low content of HPV DNA in some cervical cancers is considered as a possible cause of false negative test results. It is worth noting that the dedifferentiation and subsequent loss of HPV in the tumor may also change the HPV detection results (27).

In addition, many other factors may also be influencing factors that have no significant correlation between HPV infection and oncological outcome of IA1~IIA1 cervical cancer patients after abdominal surgery. The sample size is not large enough, the definition of HPV infection status (HPV positive cases, HPV16 positive cases or other reference categories) is different, the treatment plans received by cervical cancer patients are different, the statistical definition of survival rate is different, and there are many relative confounding factors in the actual clinical treatment process.

Real-world research has garnered increasing attention in recent times, as exemplified by the “Basic Considerations for Real-World Evidence Supporting Drug Research and Development” issued by Chinese State Drug Administration in May 2019. Although treatments were not standardized, this report represents the status of cervical cancer diagnosis and treatment in China. Moreover, this study adopted PSM to eliminate baseline heterogeneity between groups. Crucially, this study more realistically reflected the treatment status and oncological outcomes of Chinese patients with HPV-negative and HPV positive IA1–IIA2 cervical cancer, providing evidence that may not be available from randomized controlled trials.

This study has several limitations that stem from the retrospective nature of data collection. Although patients were matched based on perioperative factors to minimize bias, unknown confounding factors not captured in the dataset may have created residual bias in the results. Further, this study only focused on the analysis of survival outcomes of treatment groups with different HPV conditions after laparotomy for cervical cancer, and did not analyze the impact of specific conditions on the oncological outcome in postoperative radiotherapy, chemo-therapy and follow-up treatment. We look forward to a multicenter prospective study with a larger sample and a longer follow-up time.

## Conclusions

In conclusion, HPV-negative cervical precancerous lesions are not common in clinical practice, and their clinical characteristics and prognosis are not more favorable than those of HPV positive lesions. This study explored the impact of HPV infection on oncological outcomes of early cervical cancer by assessing patients with stage IA1-IIA2 cervical cancer undergoing surgery in parts of mainland China, encompassing 37 different regions, grades, and categories in China. This multicenter study based on real-world research contributes to previous gaps in the literature, as we provide novel insight into oncological outcomes after treatment for HPV-negative and HPV positive stage IA1–IIA2 cervical cancer in China. We aim to conduct further research in this area in order to provide a theoretical basis and novel ideas for individualized and differentiated treatment of different types of cervical lesions.

## Data availability statement

The raw data supporting the conclusions of this article will be made available by the authors, without undue reservation.

## Ethics statement

The studies involving humans were approved by The Southern Hospital Ethics Committee of Southern Medical University (Ethics No. NFEC-2017-135). The studies were conducted in accordance with the local legislation and institutional requirements. The participants provided their written informed consent to participate in this study.

## Author contributions

MH: Writing – review & editing. XS: Writing – original draft, Writing – review & editing. PaL: Writing – original draft. HZ: Writing – original draft. LS: Writing – original draft. WW: Writing – original draft. SJ: Writing – original draft. HW: Writing – original draft. PiL: Writing – review & editing. CC: Writing – review & editing.
